# BMI-1 Expression Heterogeneity in Endometriosis-Related and Non-Endometriotic Ovarian Carcinoma

**DOI:** 10.3390/ijms22116082

**Published:** 2021-06-04

**Authors:** Ludmila Lozneanu, Raluca Anca Balan, Ioana Păvăleanu, Simona Eliza Giuşcă, Irina-Draga Căruntu, Cornelia Amalinei

**Affiliations:** 1 Department of Morpho-Functional Sciences I–Histology, Pathology, “Grigore T. Popa” University of Medicine and Pharmacy, 700115 Iasi, Romania; ludmila.lozneanu@umfiasi.ro (L.L.); raluca.balan@umfiasi.ro (R.A.B.); simonaelizagiusca@gmail.com (S.E.G.); cornelia.amalinei@umfiasi.ro (C.A.); 2 Department of Pathology, “Sf. Spiridon” County Clinical Emergency Hospital, 700111 Iasi, Romania; 3 Department of Pathology, “Elena Doamna” Obstetrics and Gynecological Hospital, 700398 Iasi, Romania; 4 Department of Mother and Child Medicine, “Grigore T. Popa” University of Medicine and Pharmacy, 700115 Iaşi, Romania; ipavaleanu@gmail.com; 5 Department of Pathology, “Dr. C. I. Parhon” University Hospital, 700503 Iasi, Romania; 6 Department of Histopathology, Institute of Legal Medicine, 700455 Iasi, Romania

**Keywords:** ovarian cancer, endometriosis, BMI-1, epithelial tumor cells, stroma

## Abstract

BMI-1 is a key component of stem cells, which are essential for normal organ development and cell phenotype maintenance. BMI-1 expression is deregulated in cancer, resulting in the alteration of chromatin and gene transcription repression. The cellular signaling pathway that governs BMI-1 action in the ovarian carcinogenesis sequences is incompletely deciphered. In this study, we set out to analyze the immunohistochemical (IHC) BMI-1 expression in two different groups: endometriosis-related ovarian carcinoma (EOC) and non-endometriotic ovarian carcinoma (NEOC), aiming to identify the differences in its tissue profile. Methods: BMI-1 IHC expression has been individually quantified in epithelial and in stromal components by using adapted scores systems. Statistical analysis was performed to analyze the relationship between BMI-1 epithelial and stromal profile in each group and between groups and its correlation with classical clinicopathological characteristics. Results: BMI-1 expression in epithelial tumor cells was mostly low or negative in the EOC group, and predominantly positive in the NEOC group. Moreover, the stromal BMI-1 expression was variable in the EOC group, whereas in the NEOC group, stromal BMI-1 expression was mainly strong. We noted statistically significant differences between the epithelial and stromal BMI-1 profiles in each group and between the two ovarian carcinoma (OC) groups. Conclusions: Our study provides solid evidence for a different BMI-1 expression in EOC and NEOC, corresponding to the differences in their etiopathogeny. The reported differences in the BMI-1 expression of EOC and NEOC need to be further validated in a larger and homogenous cohort of study.

## 1. Introduction

Ovarian cancer (OC) is a gynecological malignancy that commonly originates from the ovaries, fallopian tubes, and peritoneum [1] and is considered as the most lethal malignancy with a high rate of chemoresistance and relapses. Regarding their histology, 90% of ovarian tumors are of the epithelial type [2]. 

Endometriosis represents a precursor lesion for certain types of epithelial OC, being related to microenvironment changes (such as estrogen production and dependency, progesterone resistance, and inflammation), which lead to genetic alterations and/or genetic susceptibilities that favour endometriosis-associated ovarian carcinogenesis [2,3,4]. It has been demonstrated that ovarian endometriosis, ovarian atypical endometriosis, and endometriosis-related OC (EOC) share the same genetic alterations and express clonality, while the ovarian malignant endometriosis-associated phenotype is promoted by chronic inflammation, which provides permanent mutations and nonpermanent cytokine production [2]. The different clinicopathological features and distinct mutational statuses justify the classification of OC into EOC, represented mainly by clear cell and endometrioid subtypes, and non-endometriotic OC (NEOC) [5].

OC is commonly diagnosed in advanced stages III and IV when the tumor has a high potential of metastasis [6]. Therefore, the early detection of OC by using different biomarkers is an important clinical desideration. Concomitantly, the researchers’ interest is directed towards a deep insight into the genetic and molecular substrate of ovarian carcinogenesis, aiming not only to understand the sequence of carcinogenic events, but also to identify new potential prognostic factors and therapeutic targets. The exclusive recent list of potential candidate biomarkers includes molecules expressed by the cancer stem and stem-like cells [7], BMI-1 protein being one of them [8,9]. BMI-1 protein, a stem-like marker, represents a homologue of the *Drosophila polycomb* group of proteins, and its role is the regulation of homeotic genes expression by transcription repression [10]. The BMI-1 gene has been initially isolated as an oncogene, which cooperates with c-Myc in lymphoma experimental models [11]. It belongs to the Polycomb-group (PcG) of proteins, which are involved in axial pattern establishment, hematopoiesis, cementogenesis, and senescence [11].

Considering BMI-1’s involvement in cellular proliferation and tumor progression, this gene has been identified, as expected, in a large variety of human tumors, such as: lymphoma [12,13,14], brain [15], prostate [16], oropharynx and nasopharynx [10,17,18], breast [19,20], bladder [11], gastric [21], pancreas [22], esophagus [23], lungs [24,25], head and neck cancers [26], malignant melanoma [27], pleomorphic adenoma [28], and also displaying a prognosis value in mielodysplastic syndromes [29] and in gallbladder cancer [30]. Although its action has been initially thought to be achieved by p16 suppressor gene repression, subsequent studies have demonstrated another specific mechanism of action by intercellular adhesion pathway modulation [31]. 

Limited information is available about BMI-1 in OC, as few studies address this topic, mainly providing experimental evidences [10,32,33,34,35,36,37,38,39,40,41]. BMI-1 increased expression mirrors an early and maybe reversible event in carcinogenesis [10], suggestive for an invasive and aggressive phenotype during tumor development [10,42]. It is demonstrated that BMI-1 regulates cell cycle and promotes cell proliferation, which has self-renewal and differentiation potential [9], acts as a potential modulator of cellular adhesion in endometriotic tumor cells, and alters endometrial stromal cells by changing microenvironment interactions in OC [43]. Several results support its potential value as an independent predictor for poor outcomes [39] and as a possible new therapeutic target in chemoresistant OC [7,9,33,40,41]. 

Currently, there is a high interest in a better understanding and characterization of EOC, in an attempt to provide a different clinical and therapeutic management compared to that of NEOC. In this regard, the purpose of our study was to evaluate the immunohistochemical (IHC) BMI-1 expression in two different groups of OC (associated or not with endometriosis), aiming to identify the differences in its tissue profile. The novelty of this research consisted in a double assessment of BMI-1, in tumor epithelial cells and stromal cells, following the potentiation relationship of these two cell types in tumor progression. Nevertheless, the BMI-1 expression was correlated with clinicopathological data that offer a solid functional image of the tumor progression.

## 2. Results

### 2.1. BMI-1 Expression—Qualitative Assessment 

The qualitative evaluation showed, at a glance, a heterogeneous expression in both groups, without a specific pattern for each group. 

**Figure 1:**
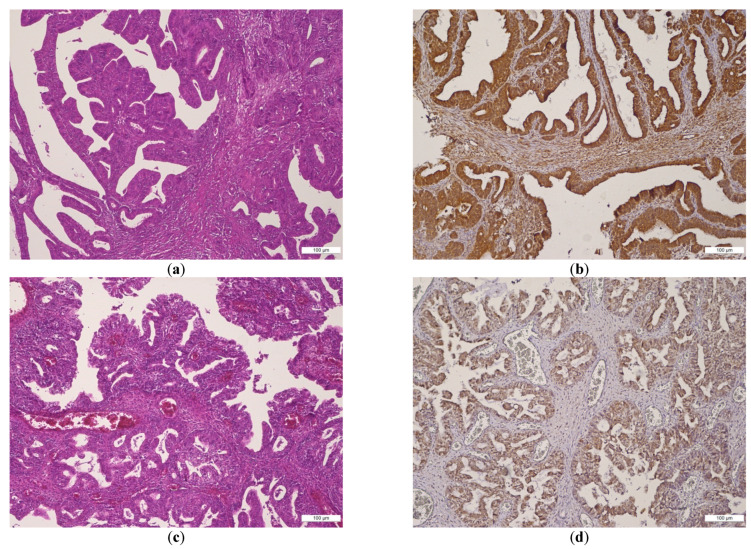

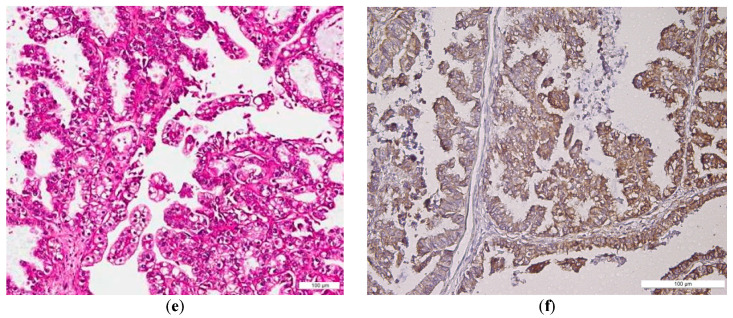
(**a**–**f**) Histologic features and BMI-1 expression in EOC group in different ovarian tumor subtypes: (**a**,**b**) HGSC: (**a**) papillary growth, enlarged and irregular nuclei, prominent nucleoli, high cellular size and shape (hematoxylin and eosin–H&E, magnification 10×), (**b**) strong BMI-1 nuclear staining in epithelial tumor cells of HGSC (magnification 10×); (**c**,**d**) HGEC: (**c**) crowded back-to-back glands, lined by atypical columnar epithelium, and smooth luminal borders (H&E, magnification 10×), (**d**) weak BMI-1 cytoplasmic staining in epithelial tumor cells of HGEC (magnification 10×); (**e**,**f**) COC: (**e**) papillary and tubulocystic pattern, combined with clear and eosinophilic cells and stromal hyalinization (H&E, magnification 10×), (**f**) strong BMI-1 cytoplasmic staining of tumor cells and stroma in COC (magnification 10×).

A double BMI-1 staining was found: a nuclear and cytoplasmic/membrane immunoexpression in EOC group. Strong expression of epithelial cells was observed in cases with poor prognosis, such as high-grade serous and endometrioid carcinomas (HGSCs and HGECs), as well in clear cell ovarian carcinomas (COCs). A negative BMI-1 stroma expression in the endometrioid phenotype of EOC group was found, while positive stroma was dominant in the serous phenotype, clear cell and mixed subtypes. Relevant aspects of BMI-1 expression in EOC are presented in Figure 1.

**Figure 2:**
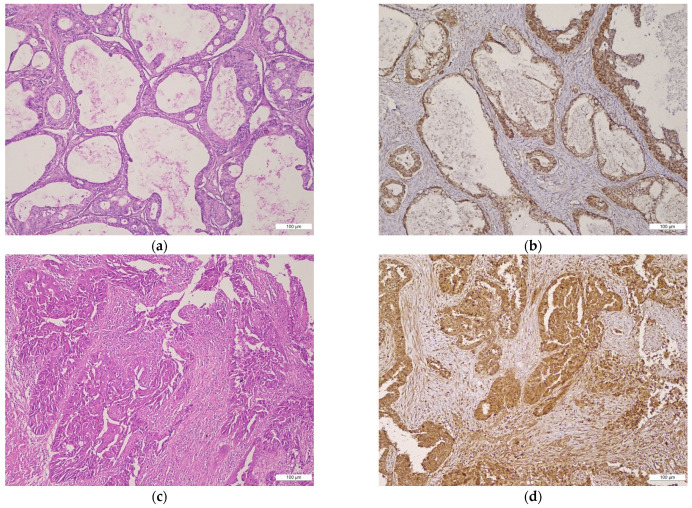

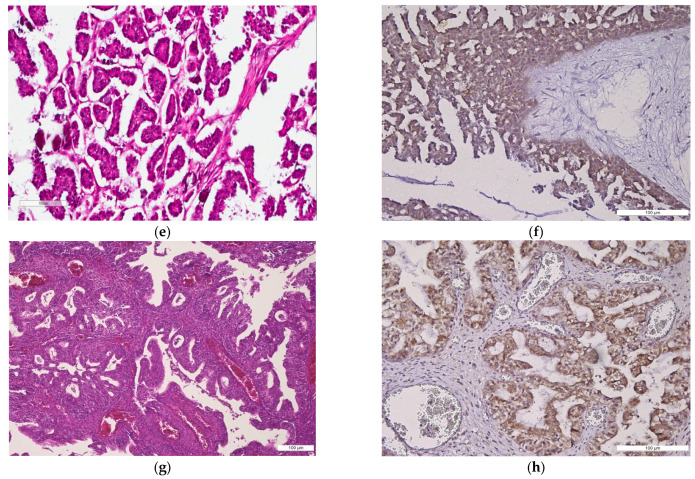
(**a**–**h**) Histologic features and BMI-1 expression in NEOC group in different ovarian tumor subtypes: (**a**,**b**) MOC: (**a**) atypical mucin-producing tumor cells with an infiltrative pattern of invasion (H&E, magnification 10×), (**b**) negative BMI-1 staining in tumor stroma of MOC (magnification 10x); (**c**,**d**) HGSC: (**c**) variation in cellular size and shape, marked nuclear atypia, dense fibrous stroma, and inflammation around the tumor nests (H&E, magnification 10×), (**d**) strong BMI-1 cytoplasmic staining of tumor stroma in HGSC (magnification 10×); (**e**,**f**) LGSC: (**e**) micropapillary growth with minimal nuclear atypia in LGSC (H&E, magnification 10×), (**f**) moderate BMI-1 cytoplasmic staining of tumor cells and stroma in LGSC (magnification 10×); (**g**,**h**) LGEC: (**g**) papillary and glandular differentiation in LGEC (H&E, magnification 10×), (**h**) strong BMI-1 cytoplasmic staining of tumor cells and stroma in LGEC (magnification 10×).

In the NEOC group, the intensity of BMI-1 was predominantly moderate or strong in epithelial (nuclear or cytoplasmic/membrane immunoexpression) and stromal cells. Moderate and strong nuclear expression and weaker cytoplasmic expression was observed in cases with a serous phenotype and a more aggressive course, such as HGSC, while the endometrioid phenotype preserved a strong, diffuse, membrane BMI-1 staining. In undifferentiated carcinomas, BMI-1 expression was heterogeneous, displaying a weak cytoplasmic staining. Differences between BMI-1 expression in variable types of NEOC are illustrated in Figure 2.

We also noted the lack of BMI-1 expression in normal ovary or ovarian surface, and its positivity in the normal tubal surface epithelium.

### 2.2. BMI Expression—Semi-Quantitative Assessment

In the whole group of study, without division into EOC and NEOC categories, the BMI-1 semi-quantitative assessment showed the following: a high expression in 31 cases (65.96%) and a low expression in 16 cases (34.04%), in tumor cells, along with immunopositivity in 34 cases (72.34%), and immunonegativity in 13 cases (27.65%) in tumor stroma. The statistical analysis revealed significant correlations between BMI-1 expression in epithelial tumor cells (low/high) versus tumor stroma (negative/positive) (*p* = 0.01).

The semi-quantitative expression of BMI-1 showed a different profile in the two analyzed groups. 

BMI-1 expression in epithelial tumor cells was mostly low or negative in the EOC group and predominantly positive in NEOC group. On the other hand, the cases of the EOC group expressed positive and negative stromal BMI-1 immunoreactions approximately equally, whereas the stromal BMI-1 expression was mainly strong in the NEOC group (Table 1). We noted statistically significant differences between the BMI-1 epithelial and stromal profiles in each group (Table 1).

Comparing the epithelial and stromal BMI-1 expressions between the EOC and NEOC groups, we obtained statistically significant differences only for the epithelial component (*p* = 0.0002), not for the stromal one (*p* = 0.06).

### 2.3. Relationship between BMI-1 Epithelial and Stromal Expression, and Clinicopathological Parameters in EOC

The results of the statistical analysis revealed a significant relationship between BMI-1 expression in tumor cells (low/high) and tumor grade (well and moderately differentiated versus poorly differentiated) (*p* = 0.04). On the other hand, stromal BMI-1 expression was significantly correlated with the median value of cancer antigen 125 (CA 125) (*p* = 0.03). No other significant differences were registered (Table 2).

### 2.4. Relationship between BMI-1 Epithelial and Stromal Expression and Clinicopathological Parameters in NEOC

The statistical analysis showed significant correlations between BMI-1 expression in the tumor cells (low/high), the stroma (negative/positive), and the tumor histological subtypes (*p* = 0.002 and *p* = 0.04, respectively) (Table 3). No associations were found for the other clinical clinicopathological parameters.

## 3. Discussion

Numerous hypotheses regarding the mechanisms involved in OC etiopathogenesis have been proposed over time as attempts to explain the multiple tumor phenotypes, poor prognosis, and chemoresistance. Endometriosis represents a precursor lesion for certain types of epithelial OC, since the identification of the same genetic alterations in both diseases are demonstrated [3,4,31]. Accordingly, the corroboration of specific clinicopathological findings with specific mutations led to the EOC and NEOC categories distinction [44].

The BMI-1 protein, involved in homeotic genes regulation by transcription inhibition [10], represents a survival factor of malignant stem cells [10], and is correlated to hormonal receptor expression, and is considered as a prognosis factor surrogate [44,45].

BMI-1 has been identified in experimental studies of OC (cell lines, clone derivation, and animal experiments) [32,33,34,35,36,37,38], both in protein and the protein-coding gene [39], and in human ovarian tumors or ascites fluid samples [10,32,34,36,39,46,47]. Despite these reported results, BMI-1 expression is not fully established in OC. The review of the literature shows that less than 10 studies have addressed BMI-1’s involvement in OC, most of them highlighting the molecular action and potential therapeutic value of this protein. A positive correlation between BMI-1 positive expression in human epithelial OC and elevated telomerase activity was demonstrated [46,47]. Another study, based on human specimens and ovarian cancer cells, showed that BMI-1 expression is downregulated by MiR-15a or MiR-16 underexpression, with subsequent significant decreases in cell proliferation and clonal growth [40]. Therefore, BMI-1 seems to be a potential target in OC therapy. Eloquent evidences in this direction are provided in recent papers that have demonstrated the therapeutic activity of PTC-028 as a novel inhibitor of BMI-1 function in OC [37] and the role of MiR-132 in cisplatin resistance and OC metastasis by the targeted regulation of BMI-1 [41]. In terms of the number of human OC samples, the studies on BMI-1 have been generally performed on small groups, with a median number of research sample of 41 (range 5–179) [10,32,34,36,40,46,47]. These samples were collected from tumor tissue [10,32,34,36,39,40,46,47], fresh ascites [34], and frozen ovarian tissues [47]. 

The reported data target only BMI-1 in epithelial tumor cells, showing a high expression in 80.9% of OC and its relationship with tumor aggressiveness [46]. Moreover, a positive correlation between BMI-1 expression and advanced International Federation of Gynecology and Obstetrics (FIGO) stages, bilaterality, higher tumor grades, and serous morphology [42,47], and a progressive incremental number of BMI-1-positive cases in accordance with the increase of tumor grade and stage were demonstrated, while increased BMI-1 expression was associated with reduced patient survival [39].

This short review of data concerning the correlation between BMI-1 and OC shows that the current knowledge is predominantly based on experimental data as the first level of evidence regarding its role in carcinogenesis, while the results obtained by the investigation of BMI-1 in human tissues is very scarce. Within this general context, our study complements the knowledge on BMI-1 in OC by doing research that translates the evidences level from the experimental area to the clinical domain by reference to the clinicopathological characteristics of OC with different parameters for EOC and NEOC.

Our work has demonstrated high BMI-1 expression levels in the epithelial tumor cells in 66% of OC (26% in EOC and 93% in NEOC). Moreover, our study provides valuable data on BMI-1 profile in OC, bringing to the foreground the relationship of OC with endometriosis, and the differences between the epithelial and stromal expression. This endeavor was possible by consistent differences in the design of the patient’s cohort, comprising 47 cases of OC separated in two different tumor groups: EOC and NEOC. Thus, we have demonstrated, for the first time, the possible correlations between epithelial and stromal BMI-1 profiles in EOC and NEOC and several classical clinicopathological parameters.

The segregation into EOC and NEOC has been justified by the findings that certain histological types of EOC, mainly endometrioid and clear cell carcinomas, have different clinical features, such as younger age at diagnosis, unilaterality, identification at an earlier stage, and a better survival rate, compared to the counterpart entities of NEOC [5]. Our study supports the hypothesis of EOC development within endometriosis, showing mostly an endometrioid (42% in EOC versus 28.57% in NEOC) or clear cell phenotype (21% in EOC versus 18% in NEOC), and, implicitly, the quality of precursor lesion of ovarian endometriosis. Endometriosis and EOC represent two entities with the same target organ (ovary), the same tissue of origin (endometrial-like), and the same pathogenic mechanism which progresses from benign to atypical and malignant phenotypes. Having these in mind, tubal ligature or salpingectomy may be used as preventative maneuvers which may be applied within a screening and early therapy algorithm.

An original finding in our research is the dual staining pattern, nuclear and cytoplasmic/membrane in both study groups, although only a nuclear staining is reported in literature [27,41,42,48]. This immunostaining pattern may indicate a possible relocation of protein during the transition to tumor phenotype. Moreover, it may suggest the involvement of additional factors as a possible reflection of adhesion molecules interrelationship in the context of epithelial mesenchymal transition (EMT) [27,48] or of the involvement of variable ovarian microenvironmental factors in both EOC and NEOC.

Our study confirms the relationship between BMI-1 in epithelial tumor cells and stroma in three instances: (i) in the general OC group (*p* = 0.01), (ii) in the NEOC group (*p* = 0.001), and (iii) in the EOC group (*p* = 0.04). In parallel, the comparative analysis of BMI-1 expression in EOC and NEOC showed a statistically significant higher expression of BMI-1 in the epithelial tumor component than in the stroma (*p* = 0.0002). Our results clearly show EOC’s association with BMI-1 low expression in epithelial tumor cells without a dominant expression profile in stromal cells, while NEOC is characterized by high BMI-1 expression in both the epithelial and stromal types of cells. However, stromal BMI-1 expression is reflecting EMT involvement in tumor progression and the interrelationship between the two cellular components, which result in BMI-1 synthesis as a stromal-dependent mechanism. Therefore, if present, stromal BMI-1 could be considered as a valuable marker for poor survival.

To the best of our knowledge, our study provides for the first time evidence for BMI-1 expression in human EOC. Differently from NEOC group findings, a progressive gain of BMI-1 expression in epithelial tumor cells has been noticed in the EOC group along with tumor grade, with statistically significant differences when we compared well and moderately differentiated with poorly differentiated tumors. This finding indicates a relationship between BMI-1 epithelial overexpression and a poorer prognosis in the selected EOC cases. Currently, CA125, expressed in the embryonic development of ovaries and re-expressed in endometriosis and ovarian neoplasms, can be used as a prognostic and predictive biomarker related to patient survival, independent of OC treatment [48]. 

CA 125 shows significant different values in the two major types of OC, suggesting that they occur as a result of different factors, following specific pathway initiations and progressions [49]. Many studies have shown that the CA125 profiles of HGSC and HGEC are different from other subtypes [50]. We also found a statistically significant correlation between stromal BMI-1 and CA 125 level, suggesting that EOC may be influenced by a microenvironment modulation specific for endometriosis-based ovarian carcinomas, supporting the rapid growth pattern and the unfavorable prognosis in a subcategory of cases. Thus, we may conclude that the interrelationship and reciprocal stimulation between a tumor’s epithelial and stromal components occurs latter during the endometriosis-related carcinogenic process, with a subsequent uptake of BMI-1 expression by stromal component, which may be reflected in an increased CA-125 level. The aggressive behavior of these EOC cases has a different significance from that of aggressive type I OC, probably originating from fallopian tube epithelium. It is worth mentioning that BMI-1 was absent in the normal ovaries or ovarian surface in the study groups, while BMI-1 expression has been identified in the normal tubal surface epithelium; this finding comports with the hypothesis of some OCs development from the fallopian tube, providing another support for this pathogenic mechanism. 

On the other hand, in the NEOC group, we have shown statistically significant differences between BMI-1 immunopositivity in the tumor’s epithelial cells, stromal cells, and histological subtypes. In our opinion, these results may be considered as solid evidence for the association of BMI-1 with high grade OC phenotypes and, consequently, with tumor aggressiveness.

Overall, our study reveals a different BMI-1 profile in the EOC an NEOC groups, thus underlying the differences in their etiopathogeny. We are aware of the limitations of our study due to the small size of the study groups and their heterogeneity in histological types, as the selection criteria have been strictly applied. Despite these limitations, our results open promising perspectives for differentiation of EOC from NEOC that need to be further validated in a larger and homogenous cohort of study. An interesting research item can be directed to the high-grade serous phenotype of OC that may be further subdivided into subcategories according to their affiliation to the EOC or to NEOC groups.

## 4. Materials and Methods

### 4.1. Patients

Our study group included 47 cases of OC, diagnosed between 2006 and 2017 and treated in several hospitals of Iasi, Romania: “Sf. Spiridon” County Clinical Emergency Hospital, “Cuza Vodă” and “Elena Doamna” Obstetrics and Gynecological Hospitals, and Oncology Regional Institute. All cases were histopathologically reassessed by two pathologists to ascertain the OC histological subtype and then divided into two groups: EOC and NEOC. The study has been approved by the Ethics Committee of “Grigore T. Popa” University of Medicine and Pharmacy, Iaşi, based on the patients’ informed consent (12378/June 2015). All subjects who provided ovarian tissue had given written and informed consent prior the surgery. 

#### 4.1.1. Clinicopathological and Tumor Serum Marker Profile of the Study Cohort

At the time of the diagnosis, the age of the patients ranged between 37 and 76 years old: 22 patients were younger (<55 years old) and 25 patients were older (≥55 years old).

Based on the standards of the FIGO staging, 17 cases were staged as FIGO stage I, 11 cases as FIGO stage II, 18 cases as FIGO stage III, and 1 case FIGO stage IV. According to tumor grade, 13 cases were graded as G1 (well differentiated), 9 cases as G2 (moderately differentiated), and 25 cases as G3 (poorly differentiated or undifferentiated). The distribution of OC histological variants was as follows: LGSC—4 cases; LGEC—5 cases; COC—9 cases; MOC—5 cases; HGSC—8 cases; HGEC—11 cases; undifferentiated—1 case; and mixed tumor (serous, endometrioid, and clear cells phenotypes)—4 cases. According to the pathogenic classification, the cases have been divided in low-grade (type I; 23 cases) and high-grade (type II; 24 cases). The histopathological exam revealed the tumor extension (residual tumor after primary surgery) in 25 cases (residual tumor ≥ 1 cm), with 13 patients diagnosed with a residual tumor < 1 cm and 9 cases without evident data about a residual tumor. Preoperatory CA125 levels higher than 35 U/mL were found in all cases comprised in the study group, ranging between 46–4163 U/mL.

#### 4.1.2. Clinicopathological and Tumor Serum Marker Profile of the EOC and NEOC Groups

In the whole group, 19 of 47 (40%) patients belonged to the EOC group and 28 patients (60%) belonged to the NEOC group. 

Cases included in the EOC group were characterized by the presence of associate endometriotic lesions consisting of the endometriosis area in the form of an endometriotic cyst lined with endometrial epithelium and endometrial stroma, as well as evidence of hemosiderin deposits and chronic hemorrhage or proliferative endometriosis foci with a well-developed glandular profile.

The mean age of patients was 59.10 ± 8.66 years in the EOC group and 56.57 ± 2.64 years in the NEOC group. The EOC group comprised the following histological types: COC—4 cases; HGSC—3 cases; HGEC—8 cases; mixed tumors—4 cases; and none of LGSC, LGEC, MOC, or undifferentiated carcinoma cases were classified as EOC. The histological types in NEOC group were: LGSC—4 cases; LGEC—5 cases; COC—5 cases; HGEC—3 cases; MOC—5 cases; HGSC—5 cases; and undifferentiated—1 case. The median value of the preoperatory CA 125 level in the EOC group was 1201.5 U/mL, while a median value of 101 U/mL was found in the NEOC group.

### 4.2. Immunohistochemistry (IHC)

The immunohistochemical staining for the identification of Antigens has been achieved using BenchMark XT automatic system (Ventana Medical System, Inc., Tucson, AZ, USA), according to protocols that needed standardization for different types of antibodies. The sections obtained from the selected paraffin-embedded blocks were dewaxed in xylene, rehydrated in ethanol, and rinsed in distillated water. The antigen retrieval was made by using the Heat-Induced Epitope Retrieval (HIER) procedure, with an antigen retrieval solution of pH 9 using CC1 solution (Ventana Medical System, Tucson, AZ, USA), consisting of a combination of ethylenediaminetetraacetic and boric acid diluted in Tris buffer for 30—60 minutes. After the endogenous peroxidase blocking with 3% hydrogen peroxide and treatment with normal goat serum 10%, used to block the non-specific protein bonds, the sections were incubated with the primary antibody BMI-1 (clone F6/ABCAM, 1/50 dilution, Abcam, Cambridge, MA, USA). Consequently, the incubation with UltraVision Quanto Detection System Horseradish peroxidase (HRP) (Igs; Ventana Medical Systems) has been performed. Antigen-antibody reaction has been visualized using 3,3`-Diaminobenzidine as a chromogen (UltraView, Ventana Medical Systems, Tucson, AZ, USA). The counterstaining of the sections was done with Mayer’s Hematoxylin. After counterstaining, the slides have been washed with liquid soap in order to eliminate the oily film, they have been rinsed with taping water and have been also bathed twice in distilled water. Negative controls have been used for results interpretation, in which primary antibodies have been skipped and replaced with distilled water and positive controls have been considered as endothelial cells and stromal fibroblasts immunostaining.

### 4.3. Semi-Quantitative Assessment

BMI-1 expression has been individually quantified in the epithelial and in the stromal components. The semi-quantitative assessment of the BMI-1 in tumor cells was done by using adapted scores based on literature reports [27,51] that took into account the staining intensity (I) and the percentage of positive cells (P). BMI-1 showed a double immunostaining, nuclear, and cytoplasmic/membrane [27,51]. The intensity of BMI-1 immunoreaction was scored as: 0—absent, 1—weak, 2—moderate, and 3—strong. The percentage of BMI-1 positive cells was scored as follows: 1—< 10%, 2—10–50%, 3—> 50%. The final BMI-1 score was obtained by multiplying P by I. BMI-1 score values < 3 were considered as a low score, and score values ≥ 3 were considered as a high score. 

For the semi-quantitative assessment of stromal BMI-1, we used a standard 2-point scale scoring system. The immunoreaction was considered negative when ≤ 10% of the tumor stromal area had a positive immunostaining of BMI-1, and positive when > 10% of the stromal area showed BMI-1 immunostaining, regardless of the level of staining intensity. 

BMI-1 expression has been independently evaluated and scored by three histopathologists with experience in immunohistochemistry interpretation and scoring differences have been revised in the evaluation panel in order to reach a consensus.

### 4.4. Statistical Analysis

Statistical analysis was carried out with Statistical Package for the Social Sciences (SPSS) v. 19 program (SPSS Inc., IBM Corporation, Chicago, IL, USA). A Chi-square (χ2) test was performed to analyze the differences in BMI-1 epithelial and stromal profile in each group and between groups, and its relationship with classical clinicopathological characteristics (age, tumor stage, grade, histological subtype, tumorigenic dualistic tumor types, residual disease, and preoperatory CA 125 level). Yates’ correction was applied when the number of cases in a subgroup was lower than five. Statistical significance was considered when *p* < 0.05.

## 5. Conclusions

Our study provides solid evidence for a different BMI-1 expression in EOC and NEOC, corresponding to the differences in their etiopathogeny. The EOCs were largely characterized by a low BMI-1 expression in epithelial tumor cells, without a dominant expression profile in stromal cells. Epithelial BMI-1 is progressively increased alongside the tumor grade and strong stromal BMI-1 may be correlated to microenvironment modulation, supporting the rapid growth pattern and the recognized poor prognosis in a subcategory of EOC cases. The NEOCs were characterized by high BMI-1 expression in both the epithelial and stromal types of cells; therefore, BMI-1 expression could be regarded as an indicator of aggressiveness of this type of malignancies in general, and for HGSC in particular. Additionally, BMI-1 expression limited to the normal tubal surface epithelium and its lack in normal germinal/surface ovarian epithelium may support the hypothesis that many OCs are originating from the fallopian tube epithelium. 

Nevertheless, the reported differences in BMI-1 expression in EOC and NEOC need to be further validated in a larger and homogenous cohort of study.

## Figures and Tables

**Table 1:** Correlations between the epithelial and stromal BMI-1 expression in the EOC and the NEOC groups.

BMI-1	EOC			NEOC		
	High Score/ Positive Reaction	Low Score/ Negative Reaction	*p* Value	High Score/ Positive Reaction	Low Score/ Negative Reaction	*p* Value
Epithelial tumor cells	5 (26.31%)	14 (73.68%)	0.04	26 (92.85%)	2 (7.14%)	0.001
Stromal cells	11 (57.89%)	8 (42.10%)		23 (82.14%)	5 (17.85%)	

**Table 2:** Correlations between BMI-1 expression in tumoral cells and clinicopathological parameters—EOC group.

Clinicopathological Characteristics	#	Tumor Cells BMI-1				*p* Value	Stromal BMI-1				*p* Value
		Low Score		High Score			Negative Reaction		Positive Reaction		
		#	%	#	%		#	%	#	%	
Age											
<55 age	8	5	62.5	3	37.5	0.34	4	50	4	50	0.55
≥55 age	11	9	81.82	2	18.18		4	36.36	7	63.64	
Tumor stage											
1	4	4	100	0	0	0.48	1	25	3	75	0.55
2	6	4	66.66	2	33.33		3	50	3	50	
3	8	5	62.50	3	37.50		3	37.50	5	62.50	
4	1	1	100	0	0		1	100	0	0	
Tumor grade											
I/II	7	7	100	0	0	0.04	4	57.14	3	42.85	0.31
III	12	7	58.33	5	41.66		4	33.33	8	66.66	
Histological subtype											
LGSC	0	0	0	0	0	0.78	0	0	0	0	0.93
LGEC	0	0	0	0	0		0	0	0	0	
COC	4	3	75	1	25		1	25	3	75	
MOC	0	0	0	0	0		0	0	0	0	
HGSC	3	1	33.33	2	66.66		1	33.33	2	66.66	
HGEC	8	6	75	2	25		5	62.50	3	37.50	
Undifferentiated	0	0	0	0	0		0	0	0	0	
Mixed	4	4	100	0	0		1	25	3	75	
Type											
Type I	4	3	75	1	25	0.94	1	25	3	75	0.57
Type II	15	11	73.33	4	26.67		7	46.67	8	53.33	
Residual disease											
NED/<1 cm	6	5	83.33	1	16.67	0.51	2	33.33	4	66.67	0.59
≥1 cm	13	9	69.23	4	30.77		6	46.15	7	53.85	
CA 125—median value											
<1201.5 U/mL	10	8	80	2	20	0.51	2	20	8	80	0.03
≥1201.5 U/mL	9	6	66.67	3	33.33		6	66.67	3	33.33	

LGSC (low-grade serous carcinoma); LGEC (low-grade endometrioid carcinoma); COC (clear cell ovarian carcinoma); MOC (mucinous ovarian carcinoma); HGSC (high-grade serous carcinoma); HGEC (high-grade endometrioid carcinoma); NED (no evident data about residual tumor).

**Table 3:** Correlations between BMI-1 expression in tumor stroma and clinicopathological parameters—NEOC group.

Clinicopathological Characteristics	#	Tumor Cells BMI-1				*p* Value	Stromal BMI-1				*p* Value
		Low Score		High Score			Negative Reaction		Positive Reaction		
		#	%	#	%		#	%	#	%	
Age											
<55 age	14	1	7.14	13	92.86	0.30	2	14.29	12	85.71	0.62
≥55 age	14	1	7.14	13	92.86		3	21.43	11	78.57	
Tumor stage											
1	13	1	7.69	12	92.30	0.91	3	23.07	10	76.92	0.71
2	5	0	0	5	100		0	0	5	100	
3	10	1	10	9	90		2	20	8	80	
4	0	0	0	0	0		0	0	0	0	
Tumor grade											
I/II	15	0	0	15	100	0.11	3	20	12	80	0.75
III	13	2	15.38	11	84.61		2	15.38	11	84.61	
Histological subtype											
LGSC	4	0	0	4	100	0.002	0	0	4	100	0.04
LGEC	5	0	0	5	100		3	60	2	40	
COC	5	0	0	5	100		0	0	5	100	
MOC	5	1	20	4	80		1	20	4	80	
HGSC	5	0	0	5	100		0	0	5	100	
HGEC	3	0	0	3	100		0	0	3	100	
Undifferentiated	1	1	100	0	0		1	100	0	0	
Mixed	0	0	0	0	0		0	0	0	0	
Type											
Type I	19	1	5.26	18	94.74	0.57	4	21.05	15	78.95	0.43
Type II	9	1	11.11	8	88.89		0	0	9	100	
Residual disease											
NED/<1 cm	16	1	6.25	15	93.75	0.83	2	12.5	14	87.5	0.72
≥1 cm	12	1	8.33	11	91.67		1	8.33	11	91.67	
CA 125—median value											
<101	14	1	7.14	13	92.86	1	4	28.57	10	71.43	0.13
≥101	14	1	7.14	13	92.30		1	7.14	13	92.86	

LGSC (low-grade serous carcinoma); LGEC (low-grade endometrioid carcinoma); COC (clear cell ovarian carcinoma); MOC (mucinous ovarian carcinoma); HGSC (high-grade serous carcinoma); HGEC (high-grade endometrioid carcinoma); NED (no evident data about residual tumor).

## Data Availability

The data used to support the findings of this study are available upon request to the authors.
